# Crizotinib in advanced non-small-cell lung cancer with concomitant *ALK* rearrangement and c-Met overexpression

**DOI:** 10.1186/s12885-018-5078-y

**Published:** 2018-11-26

**Authors:** Rui-Lian Chen, Jun Zhao, Xu-Chao Zhang, Na-Na Lou, Hua-Jun Chen, Xue Yang, Jian Su, Zhi Xie, Qing Zhou, Hai-Yan Tu, Wen-Zhao Zhong, Hong-Hong Yan, Wei-Bang Guo, Yi-Long Wu, Jin-Ji Yang

**Affiliations:** 1 Guangdong Lung Cancer Institute, Guangdong Provincial Key Laboratory of Translational Medicine in Lung Cancer, Guangdong General Hospital and Guangdong Academy of Medical Sciences, 106 Zhongshan 2nd Road, Guangzhou, 510080 China; 2 Key Laboratory of Carcinogenesis and Translational Research (Ministry of Education), Department of Thoracic Medical Oncology, Peking University Cancer Hospital and Institute, Beijing, China; 3 Department of Radiation Oncology, Xiaolan People’s Hospital Affiliated to Southern Medical University, Zhongshan, Guangdong China

**Keywords:** Anaplastic lymphoma kinase, Mesenchymal epithelial transition, Non-small-cell lung cancer, Rearrangement, C-Met, overexpression

## Abstract

**Objective:**

Crizotinib can target against mesenchymal-epithelial transition (*MET)* and anaplastic lymphoma kinase (*ALK*), which has been considered as a multi-targeted tyrosine kinase inhibitor (TKI). The objective of this study was to explore the efficacy of crizotinib in advanced non-small-cell lung cancer (NSCLC) with concomitant *ALK* rearrangement and c-Met overexpression.

**Methods:**

Totally, 4622 advanced NSCLC patients from two institutes (3762 patients at the Guangdong Lung Cancer Institute from January 2011 to December 2016 and 860 cases at the Perking Cancer Hospital from January 2015 to December 2016) were screened for *ALK* rearrangement with any method of IHC, RACE-coupled PCR or FISH. C-Met expression was performed by IHC in *ALK*-rearranged patients, and more than 50% of cells with high staining were defined as c-Met overexpression. The efficacy of crizotinib was explored in the *ALK*-rearranged patients with or without c-Met overexpression.

**Results:**

Sixteen patients were identified with c-Met overexpression in 160 *ALK*-rearranged cases, with the incidence of 10.0% (16/160). A total of 116 *ALK*-rearranged patients received the treatment of crizotinib. Objective response rate (ORR) was 86.7% (13/15) in *ALK*-rearranged patients with c-Met overexpression and 59.4% (60/101)in those without c-Met overexpression, *P* = 0.041. Median PFS showed a trend of superiority in c-Met overexpression group (15.2 versus 11.0 months, *P* = 0.263). Median overall survival (OS) showed a significant difference for *ALK-*rearranged patients with c-Met overexpression group of 33.5 months with the hazard ratio (HR) of 3.2.

**Conclusions:**

C-Met overexpression co-exists with *ALK* rearrangement in a small population of advanced NSCLC. There may be a trend of favorable efficacy of crizotinib in such co-altered patients.

## Background

Targeted therapy has led to a therapeutic paradigm shift in lung cancer, which brought the treatment of lung cancer into the era of precision medicine [1]. At least one genetic abnormality has been detected in 64% patients with lung adenocarcinoma. The rearrangement between echinoderm microtubule-associated protein like 4 (*EML4*) and anaplastic lymphoma kinase (*ALK*) resulted in the activation of *ALK* kinase, which was identified as a driver oncogene in non-small-cell lung cancer (NSCLC) in 2007 [2]. Although *ALK* fusion accounted for only 3 to 13% patients with advanced NSCLC, it has made significant effect on the treatment of advanced NSCLC as precise targeted treatment [2–4]. *ALK* rearrangement can be identified with fluorescent in situ hybridization (FISH), rapid amplification of cDNA ends coupled polymerase chain reaction (RACE-coupled PCR) and Ventana immunohistochemistry (IHC). In China, all the three methods were recommended by Chinese expert consensus and have been approved as companion diagnostic tests by the Chinese Food and Drug Administration [5].

Moreover, cellular-mesenchymal-epithelial transition (c-Met) has recently been discovered as a novel promising target in NSCLC [6]. The *c-Met* gene encodes a high-affinity receptor for hepatocyte growth factor (HGF) [7–10]. HGF binding augments the intrinsic tyrosine kinase activity of c-Met, resulted in wide range of biological activities, including cellular proliferation, motility, invasion, antiapoptotic responses, and dissemination [9]. The activation of c-Met pathway results from gain-of-function *MET* mutations, *MET* amplification and c-Met overexpression in many solid and hematological malignancies [11, 12]. *MET* amplification occurred in 7.3–10.4% of patients with untreated NSCLC [13–15]. *MET* exon 14-alteration accounted for 0.9–3.0% of lung adenocarcinoma, while c-Met protein was reportedly overexpressed in about 22.2–67.2% of NSCLC and associated with poor prognosis [16–23]. Since *MET* amplification is rare and difficulty in the detecting method of FISH, MET IHC acts as the most robust predictor of overall survival and progression-free survival to all examined exploratory markers [24]. C-Met overexpression was evaluated by IHC and a four-tier (0–3) intensity scoring system has been widely used in researches. However, a standard cutoff for c-Met overexpression has not been established yet. Patients with Met positivity of ≥50% of cells with moderate or strong staining were considered as the inclusion criteria of some clinical trials, such as MetMab clinical trials [23, 25–27], while patients with c-Met of ≥50% of cells with strong staining as the inclusion criteria of other trials [27, 28]. The activation of c-Met pathway has considered as one of the resistance mechanisms to EGFR-TKIs [29]. However, it is not clear that the role of c-Met overexpression plays in *ALK*-rearranged patients.

*ALK* rearrangements have previously been considered mutually exclusive with epidermal growth factor receptor (*EGFR*) and kirsten rat sarcoma viral oncogene homolog (*KRAS*) mutations [30–32]. However, approximately 0.3 to 1.3% of advanced patients harboring concomitant *EML4-ALK* rearrangements and *EGFR* mutations have been identified [33–35]. Similarly, overlap between *ALK* rearrangement and c-Met overexpression can also occur [20, 36]. Crizotinib, a multi-targeted TKI with activity against c-Met and *ALK*, has been considered as the first-line treatment for *ALK*-rearranged advanced NSCLC patients with remarkable response in a series of clinical trials [37–40] .The objective response rate (ORR) and median progression free survival (PFS) of crizotinib were 74% and 10.9 months in *ALK*-rearranged patients, compared with 45% and 7.0 months of chemotherapy respectively [39].

Of 19 patients with c-Met overexpression treated with crizotinib, 11 achieved partial response (PR) [41]. However, few data was available about the clinical activity of crizotinib for patients with *ALK* rearrangement and c-Met overexpression (*ALK*/c-Met) co-existence [36, 42]. Thus, our study was retrospectively conducted to investigate the frequency of *ALK*-rearrangement co-existing with c-Met overexpression in advanced NSCLC, as well as the efficacy of crizotinib for such co-altered patients.

## Methods

### Patients

This retrospective study was conducted on 4622 advanced patients from two institutes, including a cohort of 3762 patients with NSCLC at the Guangdong Lung Cancer Institute, the Guangdong General Hospital from January 2011 to December 2016 and a total of 860 at Peking University Cancer Hospital from January 2015 to December 2016. The inclusion criteria were: (1) pathologically confirmed advanced NSCLC with at least one measurable lesion; (2) identified with *ALK* rearrangement with any of these 3 methods: FISH, RACE-coupled PCR and Ventana IHC; (3) sufficient tissue for the analysis of c-Met IHC. Patients’ clinical and treatment information were extracted from electronic medical records at the Guangdong Lung Cancer Institute and the Peking University Cancer Hospital. Tumor histology was assessed by pathologists and staging was classified based on American Joint Committee on Cancer 7th edition of tumor, node, metastasis staging criteria [43]. This study was approved by the Institutional Review Board of the Guangdong General Hospital and all patients provided written informed consents.

### Study design

**Fig. 1:**
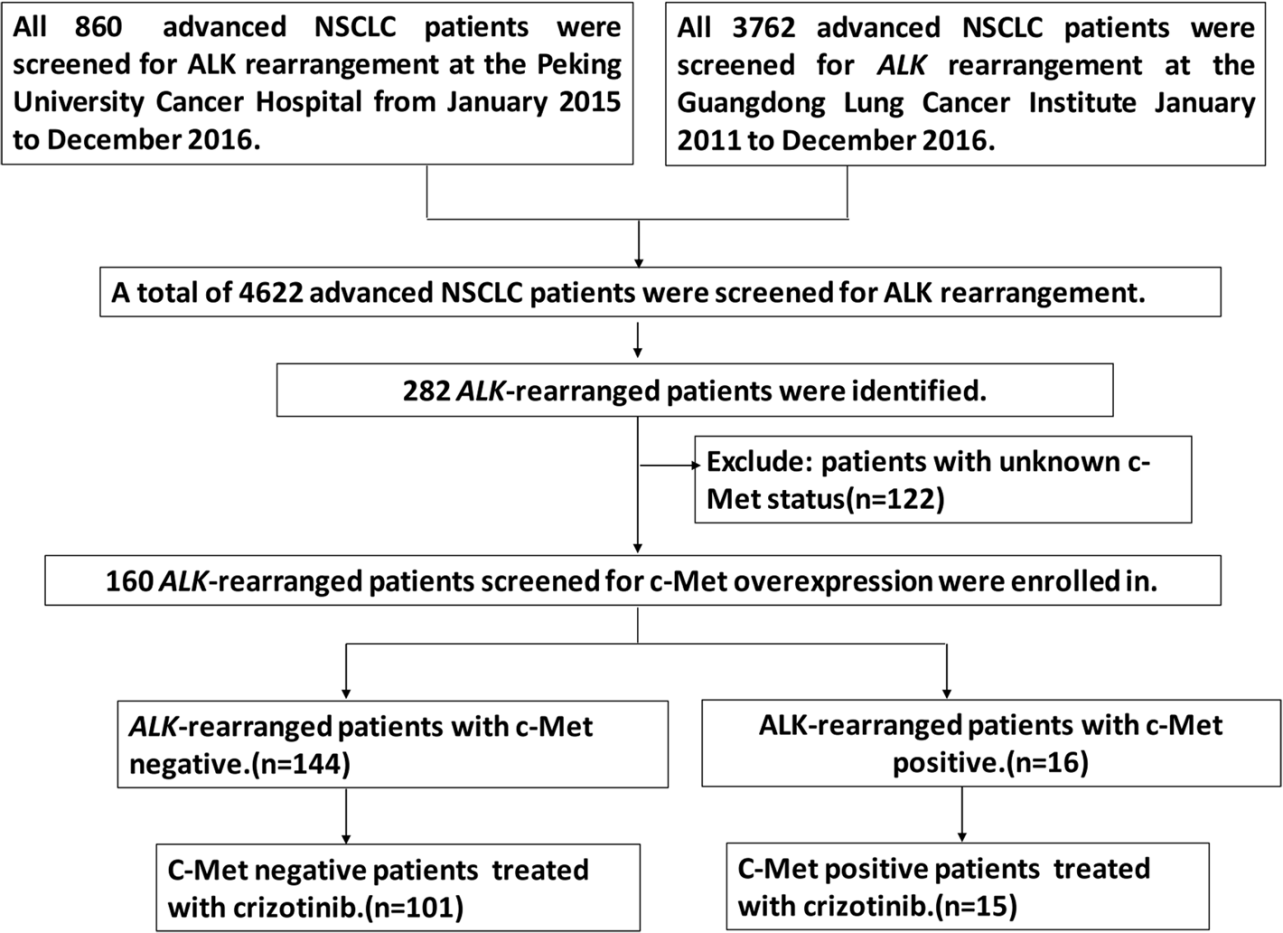
Study flow chart

In the present study, 160 *ALK*-rearranged patients were enrolled for the analysis of c-Met expression (Fig. 1). A total of 116 *ALK*-rearranged patients were treated with crizotinib. According to the results of c-Met overexpression, we divided this cohort into 2 groups: patients with c-Met overexpression and without c-Met overexpression. Objective responses were evaluated every 6 to 8 weeks according to Response Evaluation Criteria In Solid Tumors (RECIST) [44]. PFS was defined as time between the start of the treatment of crizotinib and disease progression or death. OS was measured as the period from the date of diagnosis to death resulting from any cause or censored at the last follow-up date. The last follow-up date was on October 30, 2017.

### *ALK* rearrangement analyses

All tissue samples were routinely assessed with sectioning, hematoxylin and eosin staining, and visualization with a microscope to ensure tumor content was at least 50%. IHC was carried out on 4-mm thick slides using Ventana anti-*ALK* (D5F3) rabbit monoclonal primary antibody (Roche Diagnostics, Mannheim, Germany) according to the manufacturers’ instructions. *ALK* FISH analysis was performed on formalin-fixed paraffin-embedded tissues using a commercially available *ALK* probe (Vysis LSI *ALK* Dual Color, Break Apart Rearrangement Probe; Abbott Molecular, Abbott Park, IL) according to the manufacturer’s instructions. Patients were diagnosed with *ALK* FISH-positive when 15% or more of scored tumor cells had split *ALK* 5′ and 3′ probe signals or had isolated 3′ signals. Total RNA was extracted from tissue samples with the RNeasy Kit (Qiagen). Reverse-transcriptase PCR and 5′ rapid ampli6fication c-DNA ends coupled PCR plus sequencing was performed out as ever reported [33]. PCR products were sequenced using a 3730XL Genetic Analyzer (Applied Biosystems). Target sequences were aligned with the *ALK* reference sequence (NM_004304.3) to determine if a novel fusion was present.

### Detection of c-Met overexpression and *MET* amplification

**Fig. 2:**
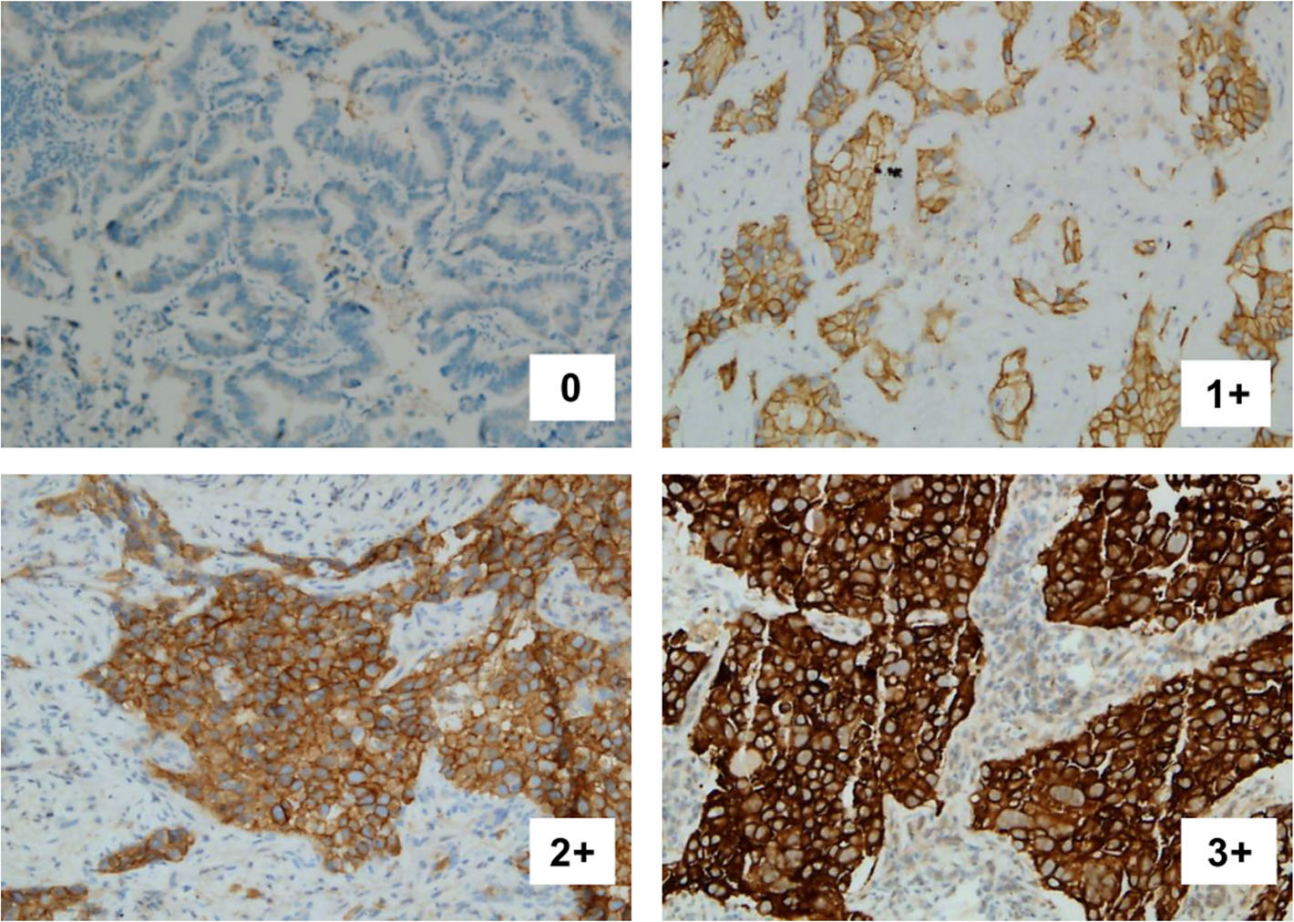
Representative examples of c-Met IHC staining with score 0 to 3 +  using MET rabbit monoclonal primary antibody (SP44).The pattern of immunostaining was mainly cytoplasmic (original magnification, × 100)

C-Met protein expression was evaluated by IHC. Sections of 4 μm thick were cut from paraffin tissue blocks of NSCLC tumors. Staining was performed on a Ventana Benchmark XT automated immunostainer (Ventana) with a CONFIRM anti-total c-Met rabbit monoclonal primary antibody (SP44, Ventana Medical Systems, Tucson, AZ, USA) and an ultra View Universal DAB. A standard protocol for immunostaining of samples was used. A four-tier (0–3) intensity scoring system has been used in our present study, as shown in Fig. 2. More than 50% of cells with high staining were considered as c-Met overexpression. *MET* amplification was detected with the method of FISH. Dual-color FISH was performed in deparaffinized sections 4 μm thick using a c-Met/CEN7q Dual Color FISH Probe (Vysis, Abbott Laboratories). After the immersion of tissue sections and TRIS-EDTA (pH = 8.0), sections were washed in PBS. Then they were digested in a protease solution at 37 °C for 7–8 min and washed in PBS once more. The sections were co-denatured with probe at 80 °C for 5 min and then hybridized at 37 °C for 14–18 h and subsequently counterstained with DAPI. The results of positivity were assessed according to the 2 criteria: A c-Met: centromere 7 ratio ≥ 2.0 and the criterion of Cappuzzi (positivity: a mean of ≥5 copies per cell, or clustered gene amplification evident in all nuclei) [45].

### *EGFR* and *KRAS* mutation analysis

Genomic DNA from each sample was used for sequence analysis of *EGFR* exons 18 to 21 and *KRAS* exons 2 and 3. These exons were amplified by PCR as previously described [46], and the resulting PCR products were purified and labeled for sequencing using the BigDye 3.1 Kit (Applied Biosystems, Foster City, CA, USA) according to the manufacturer’s protocol.

### Statistical analyses

Categorical variables were compared using X^2^ test, or Fisher exact test when necessary. Survival curves were constructed with the Kaplan–Meier method and differences analyzed by the log-rank test. Multivariable predictors were assessed by a forward step-wise likelihood ratio Cox proportional hazards model. Statistical analysis was performed using SPSS version 22.0 software (IBM, Armonk, NY). All statistical tests were two-sided, with *P* value < 0.05 considered statistically significant.

## Results

### Patients characteristics

A total of 4622 advanced NSCLC patients from two institutes (3762 patients at Guangdong Lung Cancer Institute from 2011 to 2016 and 860 cases at Perking Cancer Hospital from 2015 to 2016) with complete electronic records were screened for *ALK* gene status, of whom 282 were identified with *ALK* rearrangement. The study recruited 160 *ALK*-positive advanced NSCLC patients with sufficient tissue for the detection of c-Met expression. Sixteen patients were identified with c-Met overexpression, accounted for 10.0% (16/160) of *ALK*-rearranged patients. A total of 116 patients treated with crizotinib were investigated for the response to crizotinib. Baseline characteristics are presented in Table 1. The characteristics of *ALK*-rearranged patients with c-Met overexpression were relatively young with less than 45 years old, and most of them were female and non-smokers with adenocarcinoma. The baseline clinicopathologic characteristics were well balanced between *ALK*-rearranged patients with c-Met overexpression and those without c-Met overexpression. *MET* amplification was identified as negative by FISH in 8 *ALK*-rearranged NSCLC patients with c-Met overexpression, while 7 patients were unknown due to insufficient tissue (Table 2).

**Table 1:** Baseline clinicopathologic characteristics in *ALK*-rearranged patients with advanced non-small-cell lung cancer treated with crizotinib

Characteristic	No. of Patients (%)		*P*-value
	*ALK* + c-Met- (*n* = 101)	*ALK* + c-Met+ (*n* = 15)	
Age, years			
Median	50	44	0.551
Range	25–79	24–75	
Gender			
Male	45 (44.6%)	7(46.7%)	1.000
Female	56(55.4%)	8(53.3%)	
ECOG PS			
0–1	88(84.9%)	15(100.0%)	0.213
≥ 2	13(15.1%)	0(0.0%)	
Smoking status			
Non-smoker	83(82.2%)	14(93.3%)	0.460
Smoker	18(17.8%)	1(6.7%)	
Histology			
Adenocarcinoma	98(97.0%)	14(93.3%)	0.228
Others	3(3.0%)	1(6.7%)	
Clinical staging			
IIIB	5(5.9%)	0(0.0%)	1.000
IV	96(94.1%)	15(100.0%)	
Lines of crizotinib			
1st-line	52(51.5%)	7(46.7%)	0.787
2nd-or further-lines	49(48.5%)	8(53.3%)	

*Abbreviations*: *ALK* anaplastic lymphoma kinase, *c-Met* cellular-mesenchymal-epithelial transition, *ALK + c-Met+* patients with *ALK* rearrangement and c-Met overexpression positive, *ALK + c-Met-* patients with *ALK* rearrangement and c-Met overexpression negative, *ECOG PS* Eastern Cooperative Oncology Group performance status. Cinical staging was classified based on American Joint Committee on Cancer 7th edition

**Table 2:** The clinicopathologic characteristics and genetic profiles for patients with *ALK* rearrangement and c-Met overexpression

Patients		P1	P2	P3	P4	P5	P6	P7	P8	P9	P10	P11	P12	P13	P14	P15
Age (y)		< 60	< 60	< 60	< 60	< 60	< 60	< 60	< 60	< 60	> 60	> 60	< 60	< 60	> 60	< 60
Histology		ADC	ADC	ADC	ADC	ADC	ADC	ADC	ADC	ADC	ADC	ADC	ADC	ADC	ADC	ADC
Stage		IV	IV	IV	IV	IV	IV	IV	IV	IV	IV	IV	IV	IV	IV	IV
Smoking		0	0	0	0	0	0	0	0	0	0	0	1	0	0	0
PS		0	1	1	1	1	1	1	1	1	1	1	0	1	1	1
C-Met	IHC	50%+++, 30%+	70%+++,10%++,20%+	60%+++, 40%++	70%+++,30%+	100%+++	50%+++, 50%++	60%+++, 40%++	60%+++, 40%++	100%+++	50%+++ 50%++	90%+++	90%+++	90%+++	90%+++	90%+++
	FISH	–	–	–	–	–	ND	–	ND	–	–	ND	ND	ND	ND	ND
ALK	IHC	+	ND	+	ND	ND	ND	+	+	ND	+	+	+	+	+	+
	FISH	ND	60%	ND	29%	20%	74%	ND	ND	60%	ND	ND	ND	ND	ND	ND
	Race-PCR	ND	ND	ND	ND	V3	ND	V1	ND	ND	ND	ND	V1	ND	ND	ND
EGFR		WT	WT	WT	WT	WT	WT	WT	WT	WT	WT	WT	WT	WT	WT	19 del
KRAS		WT	WT	WT	WT	WT	WT	WT	WT	WT	WT	WT	WT	WT	WT	WT

*Abbreviations*: *F* female, *M* male, *ADC* adenocarcinoma, *PR* partial response, *m* months, *ALK* anaplastic lymphoma kinase, *c-Met* cellular-mesenchymal-epithelial transition, *EGFR* epidermal growth factor receptor, *KRAS* kirsten rat sarcoma viral oncogene homolog, *IHC* Ventana immunohistochemistry, *FISH* fluorescent in situ hybridization, *RACE PCR* rapid amplification of cDNA ends coupled polymerase chain reaction, *ND* not done, *V* variant, *19 del* exon 19 deletion

### Response to crizotinib in *ALK*-rearranged NSCLC patients with c-Met overexpression

**Fig. 3:**
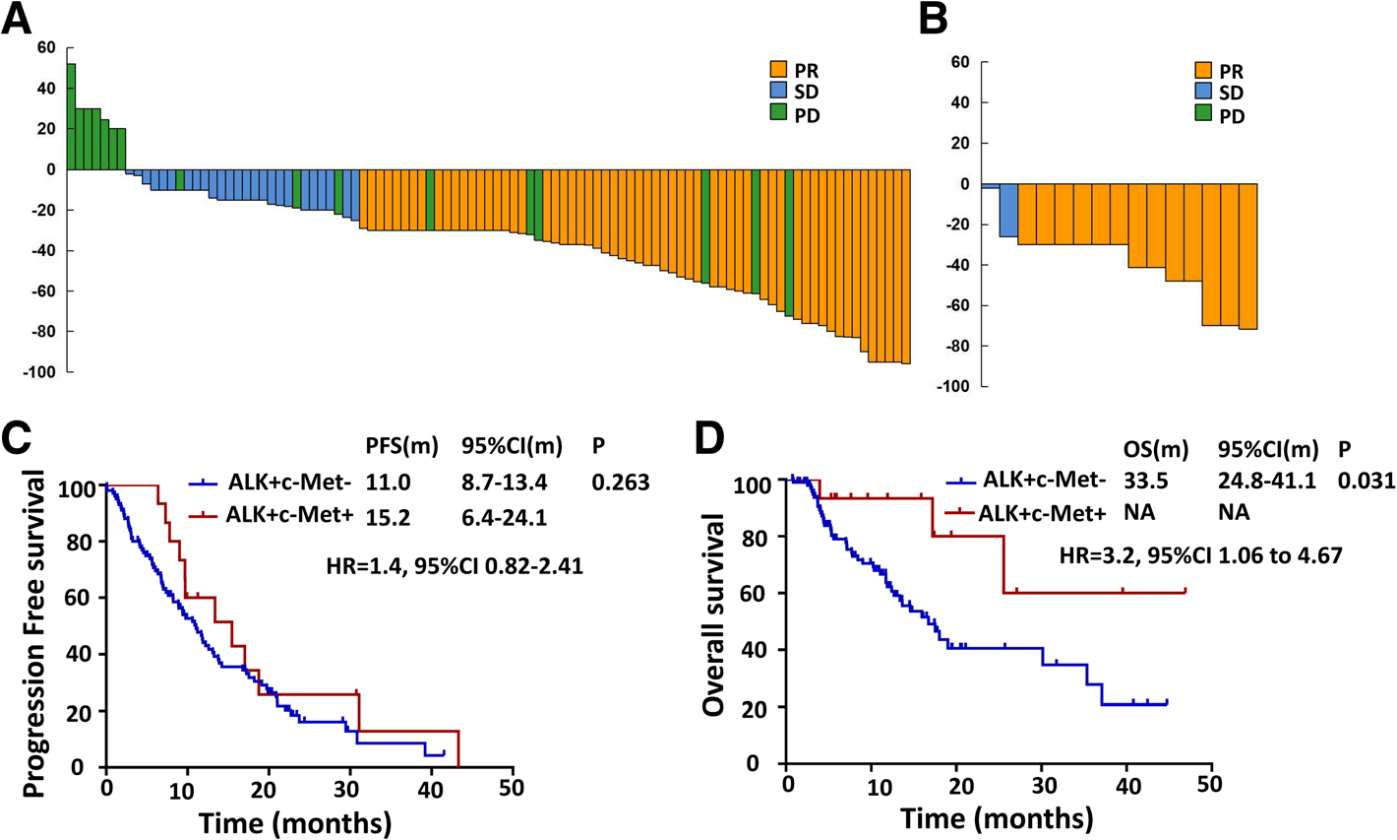
**a**, **b** Waterfall plots of the best percentage change of crizotinib in target lesions at baseline in *ALK*-positive patients with (**b**) or without c-Met overexpression (**a**). **c** PFS of crizotinib in *ALK*-positive patients with or without c-Met overexpression; **d** OS of *ALK*-positive patients with or without c-Met overexpression

A total of 116 *ALK*-rearranged patients were enrolled in our study to assess the efficacy of crizotinib, including 101 patients without c-Met overexpression and 15 cases with c-Met overexpression. Objective response rate (ORR) was 86.7% (13/15) and 59.4% (60/101) in patients with and without c-Met overexpression groups, respectively. (*P* = 0.041) (Table 3). The waterfall plots for the best percentage change in target lesion size were shown for these two groups (Fig. 3a, b).

**Table 3:** Objective response rate by crizotinib treatment

Response	No. of Patients (%)		*P*-value
	*ALK* + c-Met- (*n* = 101)	*ALK* + c-Met+ (*n* = 15)	
Complete response, n (%)	0(0.0%)	0(0.0%)	
Partial response, n (%)	60(59.4%)	13(86.7%)	
Stable disease, n (%)	25(24.8%)	2(13.3%)	
Progressive disease, n (%)	16(15.8%)	0(0.0%)	
ORR, %	59.4%	86.7%	0.041
DCR, %	84.2%	100%	0.126

*Abbreviations*: *ALK* anaplastic lymphoma kinase, *c-Met* cellular-mesenchymal-epithelial transition, *ALK + c-Met+* patients with *ALK* rearrangement and c-Met overexpression positive, *ALK + c-Met-* patients with *ALK* rearrangement and c-Met overexpression negative

### Survival in *ALK*-rearranged NSCLC patients with c-Met overexpression

Median PFS showed a trend of superiority in c-Met overexpression group (15.2 versus 11.0 months, *P* = 0.263) (Fig. 3c). The median OS was 33.5 months (95%CI, 24.8–41.1 months) in *ALK*-rearranged patients without c-Met overexpression and not estimable in those with c-Met overexpression. (HR = 3.2, 95%CI, 1.06–4.67, *P* = 0.031) (Fig. 3b). The hazard ratio for death was 3.2 (95% CI, 1.06 to 4.67). Clinical variables including gender, smoking history, pathological type, ECOG status, clinical disease stage at diagnosis were analyzed using the multivariate Cox regression model, and results indicated that ECOG status (HR = 2.6; 95% CI = 1.29–5.41, *P* = 0.008) were independent prognostic factors of OS.

## Discussion

The study of two institutes demonstrated that *ALK*-rearranged patients with c-Met overexpression that received crizotinib experienced a significant ORR, median OS and a trend of superiority of median PFS, compared with those without overexpression. It is the first study showing the clinical improvement of crizotinib for *ALK*-rearranged patients with c-Met overexpression of ≥50% of tumor cells of strong immunostaining.

The frequency of patients with c-Met overexpression were 29.2 and 58.8% of *ALK*-rearranged patients respectively in Tsuta and Feng studies, while was 10.0% (16/160) in our research [20, 36]. There were several noticeable differences between these studies and our results. Tsuta et al. defined c-Met positivity as staining in≥10% of tumor cells without concerning staining intensity, while Feng study as having ≥50% of tumor cells of immunostaining with moderate or intensity [20, 36]. However, patients with c-Met of ≥50% of tumor cells of intensity immunostaining obtained more clinical benefit from c-Met inhibitors, even achieving the ORR of 50% in several studies [28, 41]. Thus, in our study, patients with ≥50% of tumor cells of intensity immunostaining were defined as c-Met overexpression. Besides, Feng et al. study was conducted in 19 *ALK*-rearranged Western NSCLC patients with early or advanced stages, whereas ours was in 160 *ALK*-positive Eastern metastatic cases. The variations in the frequency of c-Met overexpression in *ALK*-positive patients were likely related to different definitions of c-Met overexpression and population investigated.

Crizotinib simultaneously inhibits the activation of *ALK* and c-Met pathway. *ALK*-rearranged patients obtained ORR of 74% and median PFS of 10.9 months with first-line crizotinib, while those with de novo c-Met overexpression experienced ORR of 58% [39, 41] . Thus, it is possible that *ALK*-rearranged tumors with c-Met overexpression may be shrunk largely with the treatment of crizotinib. However, only few limited cases were reported the response to crizotinib for *ALK*-rearranged patients with c-Met overexpression. Feng et al. study demonstrated 2 CRs and 4 PRs with crizotinib in 6 *ALK*-rearranged patients with c-Met overexpression [36]. However, the phase III METLung trail showed that onartuzumab plus erlotinib did not improve ORR, PFS and OS, compared with erlotinib in patients with c-Met overexpression [23]. Disease progression to crizotinib was reported in an *ALK*-rearranged patient with c-Met overexpression and Her-2 amplification [42].The clinical and therapeutic implications of c-Met overexpression underlay in *ALK*- rearranged NSCLC patients were undiscovered. Our results showed that *ALK*-rearranged patients with c-Met overexpression obtained significant objective response rate and median OS and a trend of superiority in terms of PFS with treatment of crizotinib, compared with those who without c-Met overexpression. Regardless, it would be interesting to further explore the response to the potential combination therapy of c-Met inhibitor plus *ALK* inhibitor, especially the second-generation ones, such as ceritinib or alectinib, which does not possess c-Met inhibitory activity.

However, there were several limitations in the present study. First, we focused on only a small sample-size of *ALK*-rearranged patients with c-Met overexpression. In addition, this was a retrospective study. Furthermore, the status of c-Met exon 14 skipping mutations in patients of our research was not detected for the lack of tissue samples. Finally, it is difficult to compare each *ALK* variants or other gene profiles for insufficient tissues in this study.

## Conclusion

Our study demonstrated that c-Met overexpression of ≥50% of tumor cells of strong staining exists in a small population of advanced *ALK*-rearranged NSCLC. There may be a trend of favorable efficacy of crizotinib for advanced *ALK*-rearranged patients co-existing with c-Met overexpression. Further investigations are warranted to validate our findings and to elucidate molecular mechanisms.

## Abbreviations

*ALK* + c-Met-: *ALK*-rearranged patients without c-Met overexpression
*ALK* + c-Met+: *ALK*-rearranged patients with c-Met overexpression
ALK: Anaplastic lymphoma kinase
c-Met: cellular-mesenchymal-epithelial transition
CR: Complete response
DCR: Disease control rate
ECOG: Eastern Cooperative Oncology Group
EGFR: Epidermal growth factor receptor
EGFR-TKI: EGFR-tyrosine kinase inhibitor
FISH: Fluorescent in situ hybridization
GLCI: The Guangdong Lung Cancer Institute
IHC: Ventana immunohistochemistry
KRAS: Kirsten rat sarcoma viral oncogene homolog
NSCLC: Non-small cell lung cancer
ORR: Objective response rate
OS: Overall survival
PD: Progressive disease
PFS: Progression-free survival
PR: Partial response
PS: Performance status
RACE PCR: Rapid amplification of cDNA ends coupled polymerase chain reaction
RECIST: Response evaluation criteria in solid tumors
SD: Stable disease
